# Anticancer drug discovery from Iranian *Chrysanthemum* cultivars through system pharmacology exploration and experimental validation

**DOI:** 10.1038/s41598-021-91010-y

**Published:** 2021-06-03

**Authors:** Mahboobeh Hodaei, Mehdi Rahimmalek, Mandana Behbahani

**Affiliations:** 1 Department of Agronomy and Plant Breeding, College of Agriculture, Isfahan University of Technology, Isfahan, 8415683111 Iran; 2 Department of Horticulture, College of Agriculture, Isfahan University of Technology, Isfahan, 8415683111 Iran; 3 Department of Biotechnology, College of Advanced Sciences and Technologies, University of Isfahan, Isfahan, 8174673441 Iran

**Keywords:** Cancer, Computational biology and bioinformatics, Drug discovery

## Abstract

Breast cancer is the most common carcinoma in women, and natural products would be effective preventing some side effects of cancer treatment. In the present study, cytotoxic activities of different Iranian *Chrysanthemum morifolium* cultivars were evaluated in human breast cancer cell lines (MCF-7) and human lymphocytes. A systems pharmacology approach was employed between major compounds of these cultivars (chlorogenic acid, luteolin, quercetin, rutin, ferulic acid, and apigenin) and known breast cancer drugs (tucatinib, methotrexate, tamoxifen, and mitomycin) with 22 breast cancer-related targets to analyze the mechanism through which *Chrysanthemum* cultivars act on breast cancer. Target validation was performed by the molecular docking method. The results indicated that *Chrysanthemum* extracts inhibited the proliferation of MCF7 cells in a dose- and cultivar-dependent manner. In all studied cultivars, the most effective extract concentration with the lowest viability of MCF-7 cells, was as much as 312 µg ml^−1^. Also, higher concentrations of the extracts (> 1000 µg ml^−1^) reduced the lymphocyte cell viability, demonstrating that these doses were toxic. The gene ontology analysis revealed the therapeutic effects of Chrysanthemum’s active compounds on breast cancer by regulating the biological processes of their protein targets. Moreover, it has been documented that rutin, owing to its anticancer effects and several other health benefits, is a promising multi-targeted herbal ingredient. Finally, the present study compared different Iranian *Chrysanthemum* cultivars to provide new insights into useful pharmaceutical applications.

## Introduction

Cancer, which is the second leading cause of death in the world, is the abnormal growth of tissues due to a lack of regulation in the cell division procedure^1^. Breast cancer is the most commonly diagnosed carcinoma in women^2^. Surgery, radiation therapy, chemotherapy, and targeted therapy are common methods for treating breast cancer. However, the side effects of these currently applied methods need to be minimized by developing new efficient and affordable therapies such as natural therapy. Natural products are potential multi-targeted agents that prevent associated side effects during cancer treatment^3^. About 50% of all anticancer drugs used in therapeutic trials are isolated from the natural sources related to them^4^. The medicinal value of plants depends on some phytochemicals^1^, like terpenes, phenolics, flavonoids, and alkaloids^5^. Chrysanthemum (*Chrysanthemum morifolium* Ramat.) is an important medicinal herb that has been used for centuries in many Asian countries due to its extensive biological characteristics, including its antioxidant, anticancer, antimutagenic, anti-inflammatory, and antibacterial properties^6^*.* Flavonoids, a major class of phenolic compounds, exhibit high antioxidant activity, and their regular intake would be effective for cancer prevention^7^. Recently, it has been determined that flavonoids are the main source of antioxidant activity in *Chrysanthemum* and have exhibited cytotoxic activities against human breast cancer cells. In a previous study, flavonoids extracted from *Chrysanthemum* inhibited the growth of gastric cancer cell lines and induced apoptosis in a dose-dependent manner^8^. Luteolin is considered the main reason for the antitumor activity of this plant. It inhibits the activity of topoisomerases, which are essential to the synthesis of DNA^9^. Also, it has been reported that luteolin and apigenin are two major anticancer components in vivo when *C. morifolium* is orally administrated in rats^10^. Identifying the protein targets of a bioactive molecule is the first step in developing a new drug because biomolecules exert their bioactive effects by regulating the biological processes of their protein targets^11^. Therefore, the interactions between biomolecules and their target proteins are crucial to realizing the cellular mechanisms of small molecules^12^. Using computational tools is the preferred approach to screening and confirming the therapeutic targets of active biomolecules on a large scale relatively quickly and inexpensively^13^. Previously, a computer-aided drug was designed to determine which compounds have the highest probability in pharmacological activity^14^ and molecular docking depending on the energy-based scoring function. Such work represents valuable and promising drug-discovery methods^12,15^. Herbal ingredients, which also possess a high binding affinity for breast cancer receptors, can be used in breast cancer treatment via the docking method and determine the drug-likeness of these molecules by estimating Lipinski’s Rule of Five. Lipinski’s rule, as explained by Christopher A. Lipinski (1997), is a rule used when evaluating drug-likeness. It emphasizes that an orally active drug has no more than one violation of the following criteria. For example, it has neither more than five hydrogen bond donors nor more than 10 hydrogen bond acceptors; it also has a molecular weight below 500 Daltons and a partition co-efficient log P less than 5^16^.

Considering the anticancer effect of *Chrysanthemum*, the present research uses the MTT assay to investigate the cytotoxic and growth inhibitory activities of flower extracts taken from Iranian *C. morifolium* cultivars on human breast cancer cell lines (MCF7) and peripheral blood monolayer cells (PBMC). Furthermore, a standard system pharmacology approach was applied to assess the mechanism features of *Chrysanthemum* extracts in treating breast cancer. For this purpose, we first selected and screened the major compounds of *Chrysanthemum* from our previous report^6^. Then, we utilized on-line databases to obtain the breast cancer targets and performed molecular docking to validate the targets. We also constructed a compound-target network to visualize the interactions between *C. morifolium* bioactive compounds and breast cancer-related proteins.

## Results

### Cytotoxic analysis of extracts

**Figure 1:**
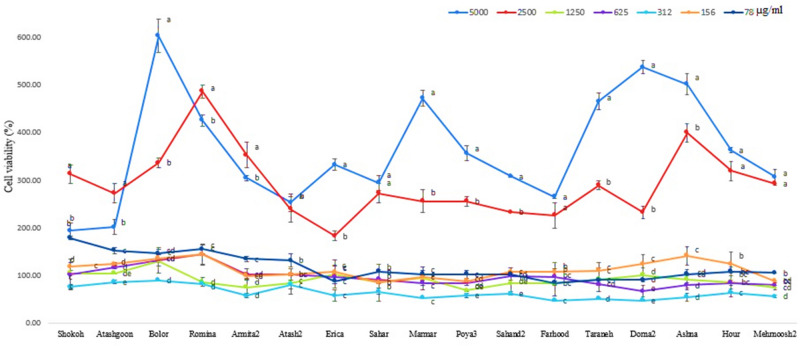
In vitro anticancer activity of the *C. morifolium* extracts against MCF-7 cell lines (Data are presented as normalized mean values of cell viabilities ± SD (n = 3).

The results showed that all tested extracts exhibited different potencies of cytotoxic activity against the MCF-7 cell lines at five different concentrations (Fig. 1). All cultivars showed maximum cytotoxic effects at a concentration of 312 µg ml^−1^, at which the methanol extracts of cultivars “Dorna2” and “Farhood” inhibited the viability of the cancer cell lines by up to 50%. In addition, these cultivars possessed higher anticancer activity than other samples at concentrations of 625 µg ml^−1^ (by more than 30%) and 78 µg ml^−1^ (15%), respectively. The “Sahand2” and “Atash2” extracts generated the greatest decreases in the viability of cancer cell lines at 1250 µg ml^−1^ (17%), while the “Sahar” extract was the most effective at 156 µg ml^−1^ (15%).

**Figure 2:**
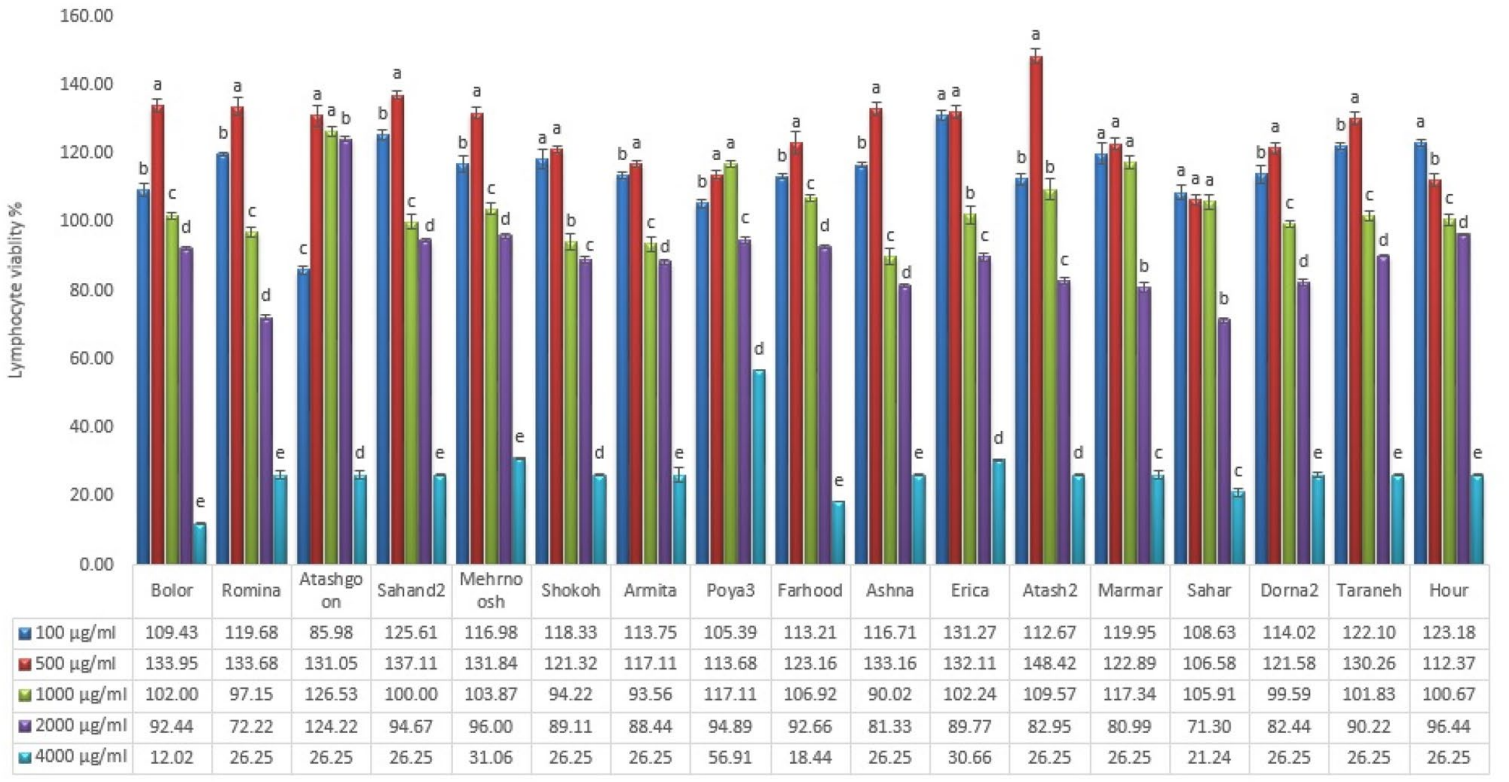
Cell viability of human lymphocyte cells against methanol extract of *Chrysanthemum* cultivars (Data are presented as normalized mean values ± SD (n = 3).

The viability of PBMC cells after exposure to all the *Chrysanthemum* extracts (100 and 500 µg ml^−1^) was greater than that of the control (Fig. 2), revealing that these doses were not cytotoxic. However, nearly all cultivar extracts increased the cell viability at 1000 mg.ml^-1^ concentration but higher concentrations (2000 and 4000 µg ml^−1^) mainly reduced the PBMC cell viability.

### Bioactive compounds and synthetic drug identification

**Figure 3:**
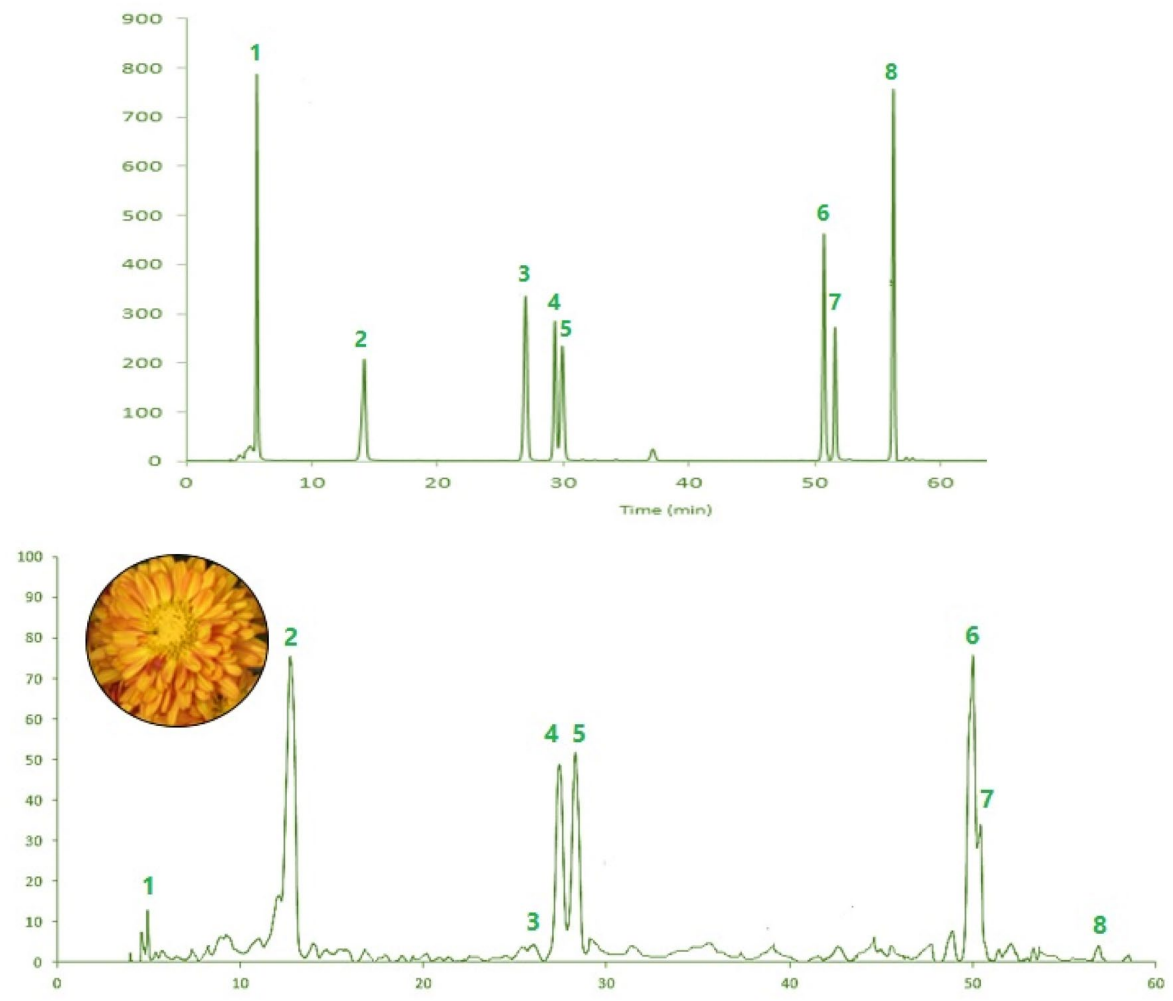
Typical HPLC chromatogram of standards and a sample (cultivar Farhood) at 270 nm. The name of each compound corresponding to each peak numbers on the HPLC chromatogram, are as follows: 1: gallic acid, 2: chlorogenic acid, 3: p-coumaric acid, 4: rutin, 5: ferulic acid, 6: luteolin, 7: quercetin, 8: apigenin.

Of the eight chemical compounds of *Chrysanthemum* obtained from our previously report^6^ (Fig. 3), six major bioactive compounds namely luteolin, quercetin, rutin, chlorogenic acid, ferulic acid, and apigenin were selected as potential active ingredients of *Chrysanthemum* cultivars. The physicochemical properties and drug-likenesses of the selected flavonoids are illustrated in Table 1.

**Table 1:** Physicochemical properties and Drug likeness of studied flavonoids.

Flavonoids	Formula	Molecular weight	Solubility in water (mg.ml^-1^)	Bioavailibility score (OB%)	Drug likeness (Lipinski)
Luteolin	C_15_H_10_O_6_	286.23	0.138	36.16	0.25
Quercetin	C_15_H_10_O_7_	302.25	0.261	46.43	0.38
Apigenin	C_15_H_10_O_5_	270.25	0.118	23.06	0.21
Ferulic acid	C_10_H_10_O_4_	194.18	0.906	40.43	0.06
Rutin	C_27_H_30_O_16_	610.51	0.125	3.20	0.68
Chlorogenic acid	C_16_H_18_O_9_	354.34	3.44	13.61	0.31

The results showed that luteolin, quercetin, and apigenin compounds passed the Lipinski rule of five parameters and satisfied the criteria of OB ≥ 30% and DL ≥ 0.18. Ingredient contents of medicinal plants should be considered in addition to the physicochemical properties of herbal compounds that influence the therapeutic effects because of their impact on the pharmacological effects of the herb^17^. Therefore three compounds, including rutin, ferulic acid, and chlorogenic acid (all of which failed to satisfy the Lipinski rule of five), were also chosen as potential ligand candidates due to their high content in some studied cultivars. Interestingly, the strong evidence about the pharmacological effects of all selected ingredients against breast cancer demonstrates the effectiveness of the proposed screening method^2,18,19^.

### Drug targeting and validation

Molecular docking was carried out to calculate the compound-target binding interactions and confirm the reliability of the selected targets (Table 2). Only those with binding free energy ≤ 5.0 kcal mol^−1^ were selected as potential targets^11^. After the docking validation process, all of 22 breast cancer-related proteins were considered as targets to be associated with *Chrysanthemum* bioactive compounds. Among the studied phytochemicals, rutin demonstrated the highest binding interaction with the most targets (12 out of 22). Luteolin was another important compound that showed the highest binding affinity (− 10.1 kcal mole^−1^); Meanwhile, ferulic acid presented the lowest binding affinity among the six tested phytochemicals (as low as − 4.9 kcal mole^−1^).

**Table 2:**
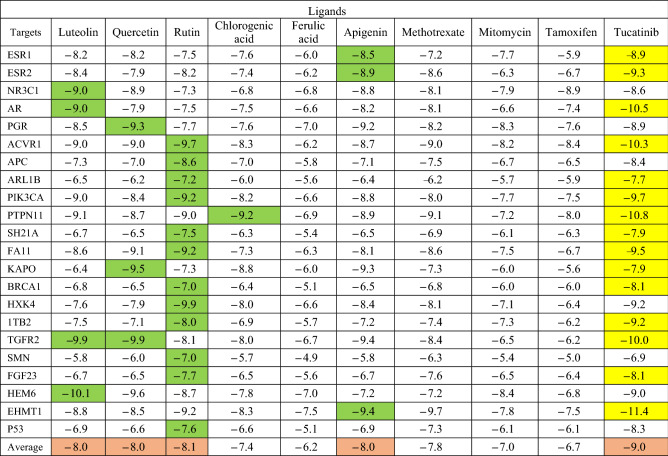
Free energy values obtained through docking analysis.

Overall, the phytochemicals of *Chrysanthemum* extracts especially rutin, luteolin, quercetin, and apigenin showed stronger interactions with different breast cancer-related targets in comparison to the three synthetic drugs (mitomycin, tamoxifen, and methotrexate). In this investigation, the ligands with the best affinity binding results (≥ − 7.0 kcal mol^−1^) were selected to establish networks between candidate ligands and breast cancer target proteins because these values suggest a strong binding affinity between the plant-compounds and proteins. Accordingly, all phytochemicals were screened out for further analysis.

### Gene ontology enrichment analysis

A gene ontology (GO) analysis was carried out to explore the functional annotation of the 22 targets of bioactive compounds and synthetic drugs. The top 10 significantly enriched GO terms were listed (Table 3).

**Table 3:** Gene ontology analysis of potential target genes.

Index	Pathway name	*p* value	Combined score
1	Breast cancer	1.560e−10	977.53
2	Estrogen signaling pathway	0.00001	296.65
3	Pathways in cancer	0.00001	112.73
4	Prolactin signaling pathway	0.00006	378.56
5	Signaling pathways regulating pluripotency of stem cells	0.0004	150.80
6	Gastric cancer	0.0005	136.97
7	Proteoglycans in cancer	0.001	89.78
8	Regulating of actin cytoskeleton	0.001	82.04
9	Endometrial cancer	0.001	197.44
10	Ras signaling pathway	0.002	72.96

GO terms with *P* value ≤ 0.01 were considered as the significant level.

**Figure 4:**
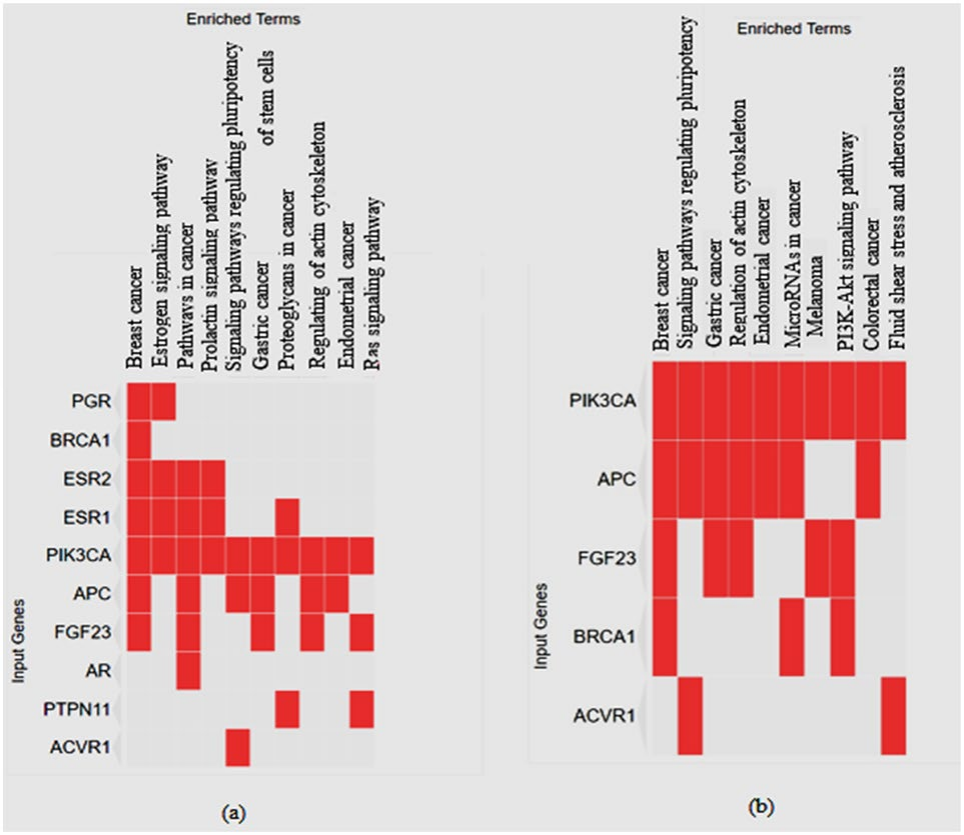
Gene ontology analysis of target genes of (**a**) six major *Chrysanthemum* compounds and (**b**) rutin.

In the present study, the top three enrichment pathways related to the biological process were breast cancer, the estrogen signaling pathway, and pathways in cancer. GO and KEGG pathway analyses showed that the four top targets (i.e., those with the most interaction with rutin), were strongly related to breast cancer. As shown in Fig. 4, several targets belong to the estrogen receptor, such as ESR1 (estrogen receptor-*α*, degree = 5), ESR2 (estrogen receptor-*β*, degree = 5), and PGR (progesterone receptor, degree = 6), are major therapeutic targets of breast cancer.

### Compound-target (C-T) network construction and analysis

**Figure 5:**
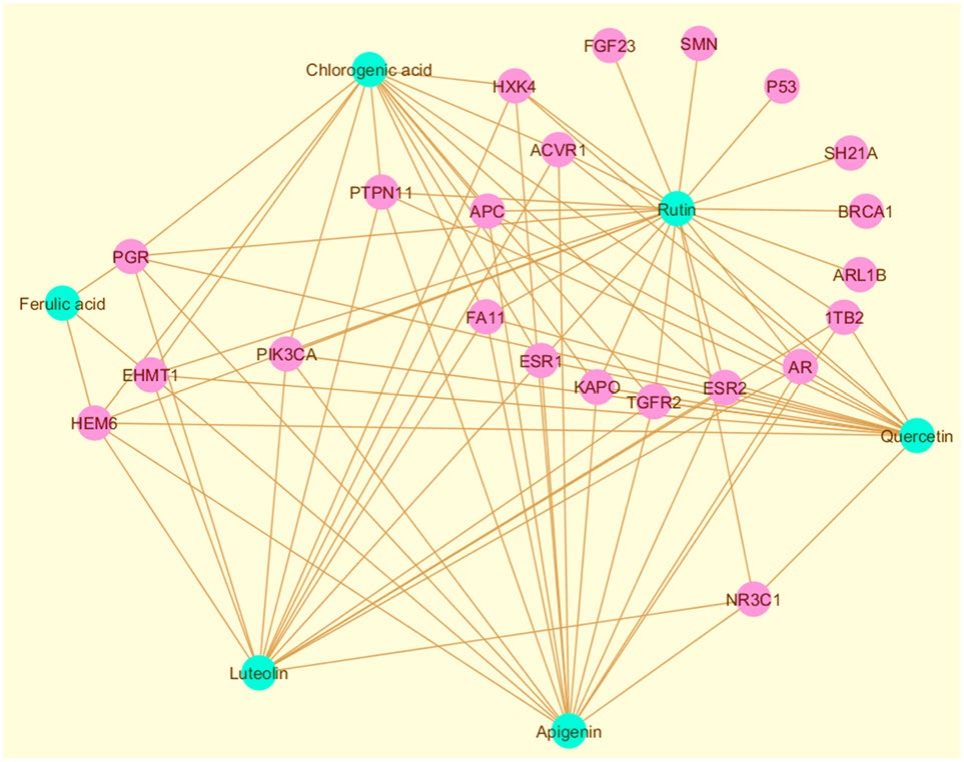
Compound-Target (C-T) network of *Chrysanthemum* bioactive compounds. Nodes represent bioactive compounds and targets.

The interaction network of 22 breast cancer-related target genes and six active ingredients of *C. morifolium* was constructed (Fig. 5). The network involved six nodes and 22 edges. The results of the CT- network revealed that rutin (degree = 22) had the highest number of interactions and is connected with all the targets, followed by apigenin, quercetin (degree = 16), luteolin (degree = 15), and chlorogenic acid (degree = 14). These single compounds can target multiple receptors confirming the multi-targeting nature of *Chrysanthemum* extract. In the present study, some targets such as PGR, HEM6, and EHMT1 were regulated by all phytochemicals (degree = 6) while some of them (ARL1B, SH21A, BRCA1, SMN, FGF23, and P53) could be regulated only with rutin (degree = 1).

## Discussion

Flavonoids possess anticancer properties due to their impact on transduction in cell proliferation^1^. Previous studies reported that the flavonoids from *C. morifolium* exhibit significant cytotoxicities against human breast, liver, and colon cancer cells^11,20^. Therefore, the observed anti-cancer effect of the extracts on the MCF-7 cell lines can be attributed to the presence of these bioactive compounds. According to the literature, both leaf and flower water extracts of *Chrysanthemum* suppressed the proliferation of MCF-7 cell lines in a dose-dependent manner at concentrations > 25 μg ml^−1^ ^21^. Another study, evaluated the anticancer activity of some Korean *Chrysanthemum* sp. (*C. boreale, C. indicum, and C. morifolium*). These methanolic extracts exhibited relatively potent inhibition at concentrations as high as 200 μg ml^−1^ against MCF-7 cell lines with cell viability of 47–63%. Treatment (200 μg ml^−1^) of *C. morifolium* suppressed 49% of cell viability in this study^22^. The protective effects of various medicinal species are crucial for their application in treating diseases such as cancer. Thus, we used the MTT assay to determine the biosafety of *Chrysanthemum* extracts on human lymphocyte cells. The results showed that high concentrations (> 1000 µg ml^−1^) largely reduced the PBMC cell viability.

The different concentrations and synergistic effects of phytochemicals in *Chrysanthemum* extracts are responsible for the variations observed in their potent anticancer activities^23^. Providing more associated pathways and molecular targets for phytochemicals is very important due to their effective role in the treatment of breast cancer^3^. High levels of estrogen are linked to an increased risk of breast cancer, which mediates its biological effects by binding to the estrogen receptor present in breast cancer cells. Flavonoids that serve as selective estrogen receptor modulators change the activities of estrogen receptors, which prevents the development of breast cancer^24^. The present study showed that targets of *Chrysanthemum* phytochemicals (luteolin, chlorogenic acid, rutin, quercetin, and apigenin) belong to estrogen receptors such as ESR1, ESR2, and PGR and are major therapeutic targets of breast cancer. Tamoxifen, as a synthetic drug for the treatment of breast cancer, binds to estrogen receptor-*α* in cancer cells, which blocks estrogen from attaching to the receptor^3^. However, greater mortality risk was observed in patients who received tamoxifen than the control group. The present research revealed that most of the investigated phytochemicals targeted breast cancer-related proteins better than tamoxifen because the anti-proliferative effect of flavonoids is selective for and sensitive to breast cancer cells^25^. The *PIK3CA* gene is one of the most frequently mutated genes in breast cancer^26^ and is expressed in most related breast cancer biological pathways. This result suggests that *PIK3CA* mutations can be considered as predictive biomarkers for breast cancer.

Targets with the highest numbers of interactions with *Chrysanthemum* phytochemicals (degree = 6) including PGR, HEM6 and EHMT1 might be major therapeutic targets for breast cancer. For example, it has been proven that PGR is an important therapeutic target in breast cancer^12^. Furthermore, the abnormal expression of EHMT1 in breast and gastric cancer was recently reported^27^. Moreover, metastasis from breast tumors can also appear as a localized lesion in the stomach, which can mimic early-stage gastric cancers^28^. Thus, *Chrysanthemum* may also play an important role in breast cancer patients by preventing breast cancer from developing into gastric cancer. Although these targets (PGR and EHMT1) of *Chrysanthemu*m have been confirmed by previous reports, HEM6’s status as a predicted target still needs to be validated by in vivo or *i*n vitro experiments.

Tucatinib as an FDA-approved targeted drug in 2020, had binding energy nearly equal to *Chrysanthemum* phytochemicals especially rutin, against most of the targets. Certain targeted therapy drugs can make hormone therapy even more effective, although these targeted drugs might also add to the side effects because they can affect other cells that have these target proteins on their surfaces^29^.

Phytochemicals exhibit pleiotropic effects and target various cancer pathways^30^. The results indicated that *Chrysanthemum* can exert its therapeutic effects by affecting multiple pathways and multiple targets along each pathway. In the present study, rutin exhibited the best binding affinity with breast cancer targets, meaning it can be effective for developing new herbal drugs that protect against breast cancer. Interestingly, rutin targets, especially APC, are also associated with other kinds of cancers such as endometrial, gastric, and colorectal cancers. Similarly, rutin has been reported to help protect against several types of cancer through various mechanisms by regulating multiple cellular signaling pathways^3^. Rutin and luteolin were the most abundant flavonoid compounds in *C. morifolium* cultivars used in the present research^6^. Among the six bioactive compounds classified as major constituents of Iranian *C. morifolium* cultivars, only three (luteolin, quercetin, and apigenin) were presented as compounds of Chinese *Chrysanthemum* in the Traditional Chinese Medicine System Pharmacology database (TCMSP). Also, the HPLC analysis confirmed the presence of luteolin, apigenin and acacetin in the Japanese *Chrysanthemum* cultivar “Kotobuki”^31^. Based on a previous report, luteolin, and diosmetin are considered anticancer flavonoids in the Chinese *C. morifolium ‘*huaiju’ cultivar. that protect against colon cancer cells^32^. Therefore, rutin is a promising anticancer agent in this plant and further research is needed to confirm its potential as an adjuvant or synergistic agent in breast cancer therapy. Among the specific targets of rutin (ARL1B, SH21A, BRCA1, SMN, FGF23, and P53), BRCA1, FGF23, and P53 were specifically related to breast cancer. Mutation of tumor suppressor gene p53 is detected in 50% of all human cancers and almost 25% of all cases of primary breast cancers^33^. Furthermore, resistance to tamoxifen in breast cancer is related to the suppression of tumor suppressor genes such as p53. As previously reported, the up-regulation of tumor suppressor (p53) by rutin and apigenin in MCF-7 cells^25^ indicates the reactivation of p53 function with significant therapeutic effects^34^. Similarly, in the present study, the cultivar “Farhood” (which presented the highest amounts of rutin and apigenin) revealed the strongest anti-cancer effect at a concentration of 312 µg ml^−1^, which was probably due to the synergistic effects of these compounds through the p53-dependent pathway. The absorption level of rutin (a rhamnoglucoside of quercetin), was one-half to one-third that of quercetin glucoside, and so it required deglycosylation by the intestinal microflora before absorption through the colon barrier^16^. Encapsulating the ingredient using nano-based drug delivery systems is a solution to overcome these challenges^35^. Previous research showed that rutin encapsulated in folic acid conjugated keratin nanoparticles significantly increased the level of ROS, which led to cell death in MCF-7 breast cancer cell lines while exhibiting less toxicity in normal cells. Furthermore, the nanoparticles enhanced the uptake of rutin in MCF-7 cells^36^. Quercetin easily passes through the colon barrier as a rutin metabolite and leads to biological effects. This could explain why rutin still has significant biological activities in in vivo models^17^. In this study, the cultivar “Taraneh” wich had the highest amount of ferulic acid, quercetin and a large amount of rutin showed high anti-cancer effects (≈ 50%) against MCF-7 cells at a concentration of 312 µg ml^−1^.

Various types of phytochemicals are present in plants. These compounds vary in molecular size, polarity, and solubility, which may affect the bioavailability and distribution of each phytochemical in different cells, organs, and tissues. The existence of a balanced indigenous combination of phytochemicals in plants cannot simply be mimicked by synthetic drugs^21^. Therefore, the use of chemo-herbal combination therapy has been reported to enhance the anticancer effects of chemotherapeutic ingredients, which is a solution to drug resistance and chemotherapy side effects^3^. Previous research has shown that a low concentration of luteolin attenuates doxorubicin-induced cytotoxicity to MCF-7 cells through a combination of antioxidant activity, as well as by increasing the levels of the anti-apoptotic protein Bcl-2^37^. Moreover, flavonoids of both rutin and apigenin synergistically enhanced tamoxifen’s anti-proliferative effect against MCF-7 cells^25^. Therefore, it is recommended to make medicines containing a combination of tucatinib with phytochemicals such as rutin, apigenin, and luteolin to improve the therapeutic effects of anticancer agents. Also, using the flower extracts of ideal *Chrysanthemum* cultivars that are rich in these compounds, such as “Farhood” and “Taraneh” cultivars alone or in combination with tucatinib, can be effective in the treatment of breast cancer.

## Conclusion

The present study highlighted the possible safe use of different Iranian *Chrysanthemum* cultivars as anticancer agents at concentration of about 300 µg ml^−1^. A strategy was applied to obtain the mechanism of *Chrysanthemum* flower extracts for treating breast cancer. Computational tools revealed that the phytochemical compounds taken from flower extracts of *Chrysanthemum* had a strong inhibitory effect on breast cancer cell lines (MCF-7 cells). This finding indicates that the anticancer activity of *Chrysanthemum* extracts was attributed to the complex mixture of phytochemicals present in these extracts. A single antioxidant could not successfully replace the combination of natural phytochemicals in health benefits. A GO analysis showed that *Chrysanthemum* compounds could also affect many disease-associated pathways, including pathways in cancer in addition to the non-disease-associated pathway*.* The study revealed that *Chrysanthemum* phytochemicals especially rutin could not only regulate breast cancer pathways but also prevent breast cancer from developing into gastric and other types of cancers. Thus, the study provides in vitro and in silico validation for the anticancer activities of *Chrysanthemum* extracts and their major flavonoids. As a result, the present research suggests that the multi-targeting nature of *Chrysanthemum* flowers, is worth further investigation to promote *Chrysanthemum* natural flavonoids as potential molecules in clinical trials.

## Methods

### Ethics statement

This study was performed in accordance with the Ethics Committee of Isfahan University, Iran. Written, informed consent was obtained from all volunteers. All experimental protocols were approved by the Ethical Committee in Isfahan University, Iran.

### Sample preparation and extraction

Sixteen cultivars of *Chrysanthemum* (*Chrysanthemum morifolium* Ramat.) including Shokoh, Atashgoon, Atash2, Sahar, Marmar, Sahand2, Dorna2, Ashna, Hour, Erica, Poya3, Bolor, Romina, Farhood, Taraneh, and Mehrnoosh2 were used in the present study. Plants were collected from the National Research Center of Ornamental Plants, Mahallat, Iran and cultivated at the experimental farm of Isfahan University of Technology on 22 April 2015 using a randomized complete block design with three replicates. The collection of these cultivars was permitted by National Institute of Ornamental Plants, Mahallat, Iran and it complies with local and national guidelines and legislation. The determination of plants was performed by Dr. Mehdi Rahimmalek from Isfahan University of Technology, Iran. The flower samples were collected in October and November during the full flowering stage when the ray florets were completely open. The flower samples were collected in October and November during the full flowering stage when the ray florets were completely open. Dried ground flowers (2.5 g) were extracted with methanol–water (50 ml, 80:20, v/v) and an orbital shaker (150 rpm) at 25 °C for 24 h. The suspension was then filtered^38^. The filtered methanolic extracts were evaporated under reduced pressure to obtain a dried residue, and a stock solution was then prepared by dissolving the extract powders in DMSO^39^ to form concentrations as high as 100 mg ml^−1^.

### Culture and maintenance of MCF-7 cell lines

Human cancer cell lines (line MCF-7) were acquired from National Cell Bank of Pasture Institute, Tehran, Iran. Cell lines were cultured in RPMI-1640 media containing 10% fetal bovine serum, 2 mM glutamine, and antibiotics (100 U ml^−1^ penicillin, 100 µg ml^−1^ streptomycin). Three wells were seeded for each concentration, and maintained in a humidified 5% CO2 incubator at 37 °C ^40^.

### Culture and maintenance of peripheral blood monolayer cell

Heparinized total blood was taken from five healthy volunteers, and a Lymphodex solution was added and centrifuged at 1800 rpm for 20 min. The human mononuclear lymphocytes were then isolated and suspended in a DMEM medium with 10% fetal bovine serum, followed by incubation in a humidified 5% CO2 incubator at 37 °C. Finally, the cells were plated in 96-well plates and used for cytotoxicity assay^41^.

### MTT assay

The effects of *Chrysanthemum* extracts on peripheral blood monolayer and MCF-7 cells were determined by 3-(4, 5 dimethylthiazol-2-yl)-2, 5- diphenyltetrazolium bromide (MTT) assay^1^. A 180 µl volume of medium containing 5000 cells was seeded in 96-well microplates. The plant extracts were then added at dilutions of 100, 500, 1000 and 2000 µg ml^−1^ in the final volume for lymphocyte cells and 5000, 2500, 1250, 625, 312, 156, 78 µg ml^−1^ in the final volume for human breast cancer cell lines. After 24 h of incubation in a humidified atmosphere of 5% CO_2_ at 37 °C, the plates were incubated for 48 h. The cell viability was determined by adding 20 µl of an MTT solution to each well, followed by incubation for 6 h. The metabolically active cells reduced MTT dye to formazan crystals. The supernatant was discarded, and 100 µl of DMSO was added to all wells. Afterward, the microplates were placed at room temperature for about 2 h to complete the dissolution of formazan. Finally, the absorbance was read at 550 nm using a microplate reader^40^. The cell viability was calculated using the following formula:

$$ \% \;{\text{Cells}}\;{\text{viability}} = \left( {{\text{OD}}\;{\text{sample/OD}}\;{\text{control}}} \right) \times 100 $$

### Ligand preparation and screening

The major components of studied *Chrysanthemum* cultivars, including luteolin, quercetin, rutin, chlorogenic acid, ferulic acid, and apigenin were retrieved from our previous report^6^. In that report, we identified the flavonoid compounds of these cultivars using High-Performance Liquid Chromatography analysis. The synthetic drugs related to breast cancer were retrieved from Drug Bank (http://www.drugbank.ca/) and used for comparisons in this process. The drug-like molecules needed to be screened based on ADME (absorption, distribution, metabolism, and excretion) properties, which are related to factors that influence the ingredients in the human body. The physicochemical properties and drug-likeness predictions of the compounds were carried out based on “Lipinski’s Rule of Five”^42^ using the Traditional Chinese Medicine Systems Pharmacology (TCMSP; http://ibts.hkbu.edu.hk/LSP/tcmsp.Php) and Drug Bank databases. Two ADME-related important parameters namely, oral bioavailability (OB) ≥ 30% and drug-likeness (DL) ≥ 0.18 were set as the threshold to select candidate active ingredients^11^. OB is an important pharmacokinetic criterion in screening bioactive molecules, as it shows whether the orally-administrated dose arrives in the human body unchanged^42^.

### Retrieval of breast cancer targets

A literature survey and the DisGeNet database (http://www.disgenet.org) were employed ^43^ to identify effective therapeutic targets related to breast cancer. Twenty-two genes with a strong or definitive evidence level of gene-diseases association (Score = 1) were selected for further analysis among the 380 potential targets entered into the DisGeNet database (Table 4).

**Table 4:** The detailed information of 22 target proteins.

Protein name	Gene name	UniProt ID
Estrogen receptor	ESR1	P03372
Estrogen receptor beta	ESR2	Q92731
Glucocorticoid receptor	NR3C1	P04150
Androgen receptor	AR	P10275
Progesterone receptor	PGR	P06401
Activin receptor type-1	ACVR1	Q04771
Adenomatous polyposis coli protein	APC	P25054
AT-rich interactive domain-containing protein 1B	ARI1B	Q8NFD5
Phosphatidylinositol4,5bisphosphate 3-kinase catalytic subunit alpha isoform	PIK3CA	P42336
Tyrosine-protein phosphatase non-receptor type 11	PTPN11	Q06124
SH2 domain-containing protein 1A	SH21A	O60880
Coagulation factor XI	FA11	P03951
cAMP-dependent protein kinase type I-alpha regulatory subunit	KAPO	P10644
Breast cancer type 1 susceptibility protein	BRCA1	P38398
Hexokinase-4	HXK4	P35557
Integrin beta-2	ITB2	P05107
TGF-beta receptor type-2	TGFR2	P37173
Survival motor neuron protein	SMN	Q16637
Oxygen-dependent coproporphyrinogen-III oxidase, mitochondrial	HEM6	P36551
Histone-lysine N-methyltransferase EHMT1	EHMT1	Q9H9B1
Cellular tumor antigen p53	P53	P04637

### Compound- target interaction validation

The 3D structures of compounds were prepared in SDF format using PubChem and the crystallographic structures of targets were obtained from the Protein Data Bank (http://www.rcsb.org/). Molecular docking approaches were applied to perform the docking process with conformations of targets and ligands through the AutoDock Vina program’s scoring functions in PyRx virtual screening software^44^. The rest of the parameters were set to default values. The conformations with 0.0 Å in positional root-mean-square deviation (RMSD) were selected. The highest (most negative) binding energy was considered as the ligand with maximum binding affinity. Crystalized ligands and solvent molecules in the 3D model target proteins were eliminated before docking and optimized by merging nonpolar hydrogen with Gasteiger charges using UCSF Chimera software. Furthermore, the SDF formats of the ligands were converted to the PDBQT format and arranged as a spreadsheet using the PyRx tool.

### Gene ontology (GO) analysis

Gene ontology (GO) analysis for breast cancer target genes was performed to determine the biological process of targets using the Clustergrammer (http://github.com/MaayanLab/clustergrammer) and the independent data visualization module. GO terms with P-value ≤ 0.01 were considered as the significant level. The pathway information was extracted from KEGG (Kyoto Encyclopedia of Genes and Genomes, http://www.genome.jp/kegg/)^45^.

### Network construction

A compound-target (C-T) network was used to visualize complex interactions between *C. morifolium* bioactive compounds and breast cancer-related proteins. This network was generated by Cytoscape (https://www.cytoscape.org/), a popular bioinformatics platform for biological network visualization and validation^11^. The network is composed of nodes and edges, which indicate molecules and intermolecular interaction, respectively. The node size corresponds to its degree.

### Data analysis

The statistical analyses were performed through one-way analysis of variance (ANOVA), and Fisher’s (protected) least significant difference (LSD) was applied to compare means at the 95% confidence level using SAS ver. 9 software. All assays were carried out in triplicate. The analysis values were expressed as mean ± standard deviation.
